# Synthesis and Properties of La_14_*TME*_6_CuS_24_O_4_, Exhibiting Multivalent Spin Chains (*TME* = Cr, Fe)

**DOI:** 10.1021/acsomega.4c00564

**Published:** 2024-05-08

**Authors:** Emil H. Frøen, Martin Valldor

**Affiliations:** Department of Chemistry, University of Oslo, Sem Sælands vei 26, NO-0316 Oslo, Norway

## Abstract

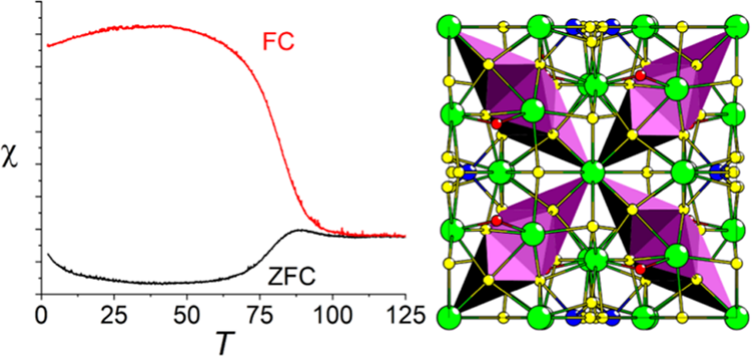

Two homologues in a series of quinary oxysulfides, La_14_*TME*_6_CuS_24_O_4_ (*TME* = Cr or Fe), have been synthesized by solid-state synthesis in sealed ampules, and their structures are homologue to assume a novel crystal structure. X-ray diffraction analyses of single crystal and powder samples give a monoclinic lattice, described in the *C*2/*m* (No. 12) space group, with lattice parameters *a* = 15.3853(5) Å, *b* = 13.9729(5) Å, *c* = 10.5074(4) Å, and β = 116.227(3)° for the Cr analogue and *a* = 15.4303(2) Å, *b* = 14.0033(2) Å, *c* = 10.4909(2) Å, and β = 116.261(2)° for Fe. The crystal structure contains one-dimensional (1D) chains consisting of interconnected transition metal element (*TME*) trimers, which are further arranged into two-dimensional (2D) layers. These spin-chain planes are interspaced with 1D chains of lanthanum–oxygen coordinations and an apparent disordered occupation of copper sites. Alternating current (AC) and direct current (DC) magnetic susceptibility measurements show that the Cr and Fe analogues exhibit what is best described as spin-domain formation. Density functional theory (DFT) calculations suggest the formal oxidation state of the species is best represented in the form La_14_*TME*_5_^2+^*TME*^3+^Cu^+^S_24_O_4_.

## Introduction

Multianion compounds, through the history of modern chemistry, have always been overshadowed by the monoanion field. One may postulate a range of reasons why this has always been the case, but a major contributing factor is likely that the cations were perceived as the source of a compound’s physical properties and that very few multianion systems are found in nature. Despite the major influence anions have on these effects, their contribution is often marginalized as a framework for the cations.

Multianion compounds may exhibit either solid-solution or superstructure arrangements, as determined by their ionic radii. According to Hume-Rothery, two anions with ionic radii that differ by less than 15% tend to form solid solutions, while greater differences will typically result in an ordered superstructure.^1^ The oxide–sulfide pair is one example that fulfills this criterion for a superstructure.^2,3^

The introduction of a second anion to a crystal structure increases the degrees of freedom a structure has in terms of assuming an ordered arrangement, allowing for arrangements and structural symmetries that would be improbable in a monoanionic compound. By extension, this gives multianionic compounds the potential to exhibit properties that cannot be achieved by monoanionics. This potential has, in recent years, caused interest in multianionic compounds to rise in fields including novel crystal structures,^2,3^ tunable properties such as band gaps,^4^ optical properties,^5^ catalysis,^6^ superconductivity,^7^ and more. Along with the wide range of unexplored phase diagrams, multianion compounds are promising grounds for chemical exploration. As a more concrete example, mixed anion compounds are promising candidates for applications within nonlinear optics in the infrared spectrum. Currently utilized materials in the infrared range suffer from intrinsic drawbacks, making improved materials an appealing prospect.^8^ Among the relevant materials investigated to date are several oxychalcogenides, including BaGeOSe_2_,^9^ CaCoSO,^10^ and SrZn_2_S_2_O,^5^ making the oxysulfides an attractive target for further investigation.

Here, we present two isostructural, novel quinary oxysulfides, La_14_*TME*_6_CuS_24_O_4_ (*TME* = Cr and Fe), and their syntheses, structures, and properties. The compounds will henceforth individually be referred to as LCCSO and LFCSO for the Cr and Fe analogues, respectively, and colloquially as LTCSO.

## Results

### Crystal Structure

**Figure 1:**
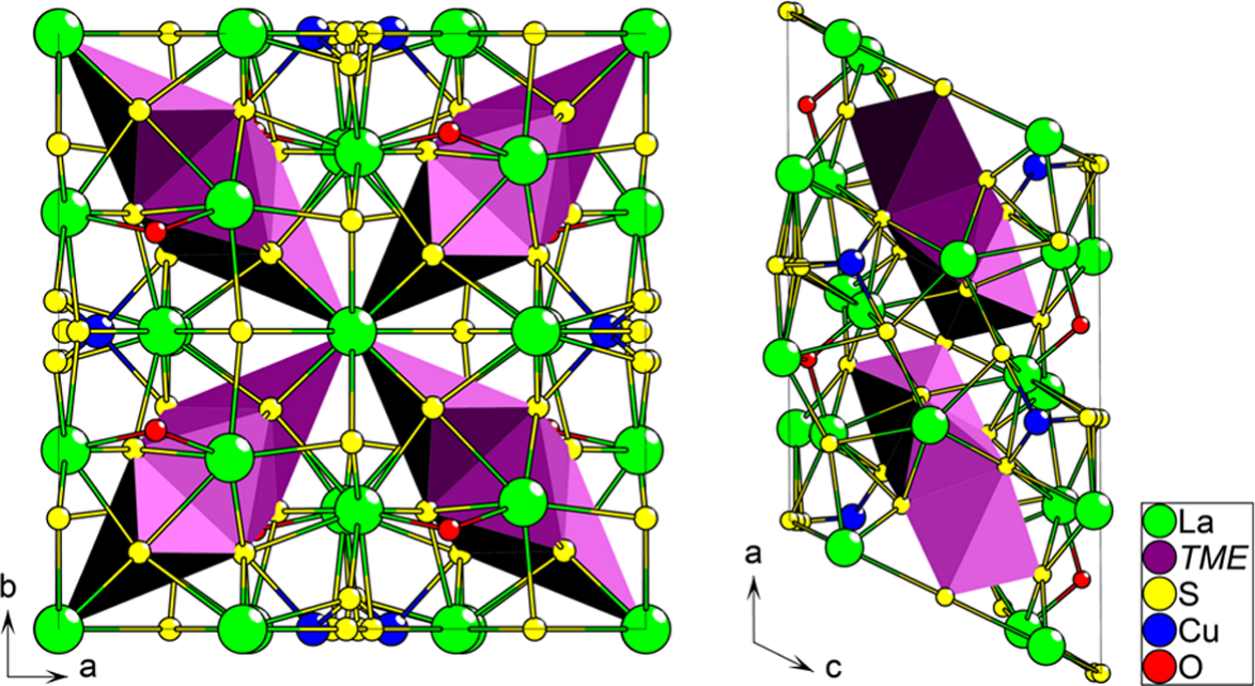
Full crystal structure of LTCSO.

The single-crystal structure determination of LTCSO shows that the phases assume a monoclinic crystal structure with space group *C*2/*m* (No. 12). The refinement data and lattice parameters for both analogues are given in Table 1. The full ionic positions and thermal parameters are given in the Supporting Information. The overall structure is shown in Figure 1.

**Table 1:** Results of the Refinement of the Structure of LTCSO against Single-Crystal X-ray Diffraction (SC-XRD) Data

formula	La_14_Cr_6_CuS_24_O_4_	La_14_Fe_6_CuS_24_O_4_
radiation	Mo Kα (λ = 0.71073 Å)	
instrument	BRUKER D8 Venture	
physical appearance	black	
crystal system	monoclinic	
space group	*C*2*/m* (no. 12)	
formula weight/g mol^–1^	3153.64	3176.75
temperature/K	293	
*a*/Å	15.376(1)	15.437(1)
*b*/Å	13.9611(9)	14.0102(9)
*c*/Å	10.4925(7)	10.4973(7)
β/Å	116.300(2)	116.307(2)
*V*/Å^3^	2035.1(2)	2035.1(2)
*Z*	2	
ρ_calc_/g cm^–3^	5.1869	5.1840
independent reflections	2629	4459
no. of variables	126	126
GOF (obs) on *F*^2^	1.99	1.63
GOF (all) on *F*^2^	1.86	1.56
*R*1 (obs)/%	5.36	2.41
*R*1 (all)/%	6.85	3.46
w*R*2 (obs)/%	18.30	5.46
w*R*2 (all)/%	18.82	5.67
CCDC ID	2 309 954	2 309 953

The structure of LTCSO (Figure 1) may be described in terms of three prominent features: The one-dimensional (1D) lanthanum-oxide chains, the partially occupied Cu positions, and the sulfide-coordinated transition metal element (*TME*) triplets are arranged into a two-dimensional (2D) network of spin chains. The preferential coordination of the oxide with the lanthanum cations is in accordance with the hard–soft-acid–base (HSAB) principle and is a common feature observed in lanthanide oxysulfides, provided there are no comparable or harder cations present in the composition.

**Figure 2:**
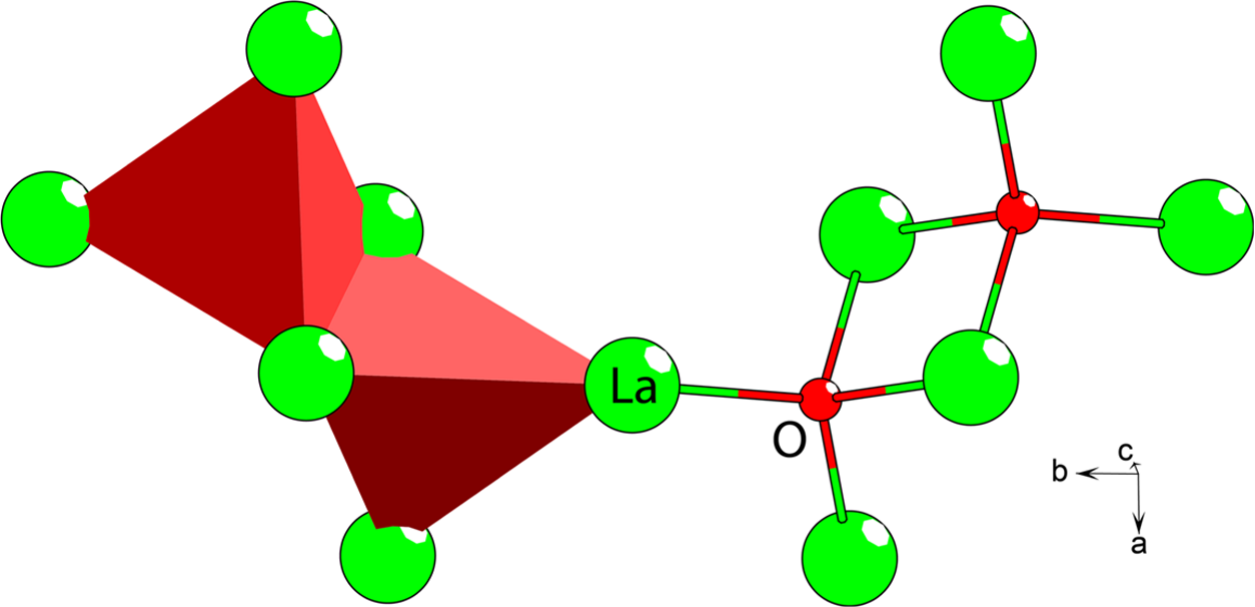
Arrangement of the lanthanum–oxygen chains.

The La–O chains are arranged with each oxide ion tetrahedrally coordinated by four La ions (Figure 2). Two adjacent [La_4_O] tetrahedra are arranged in an edge-sharing configuration, forming hourglass-shaped [La_6_O_2_] units. These larger [La_6_O_2_] units are then again arranged into a mirrored vertex-sharing arrangement with their adjacent equivalents, forming the final 1D chains, which are arranged parallel with the *b*-axis. The adjacent 1D chains are further arranged into a pseudoplanar arrangement, interspaced with the *TME* layers along the *c*-axis, and the copper positions along *a*-axis.

**Figure 3:**
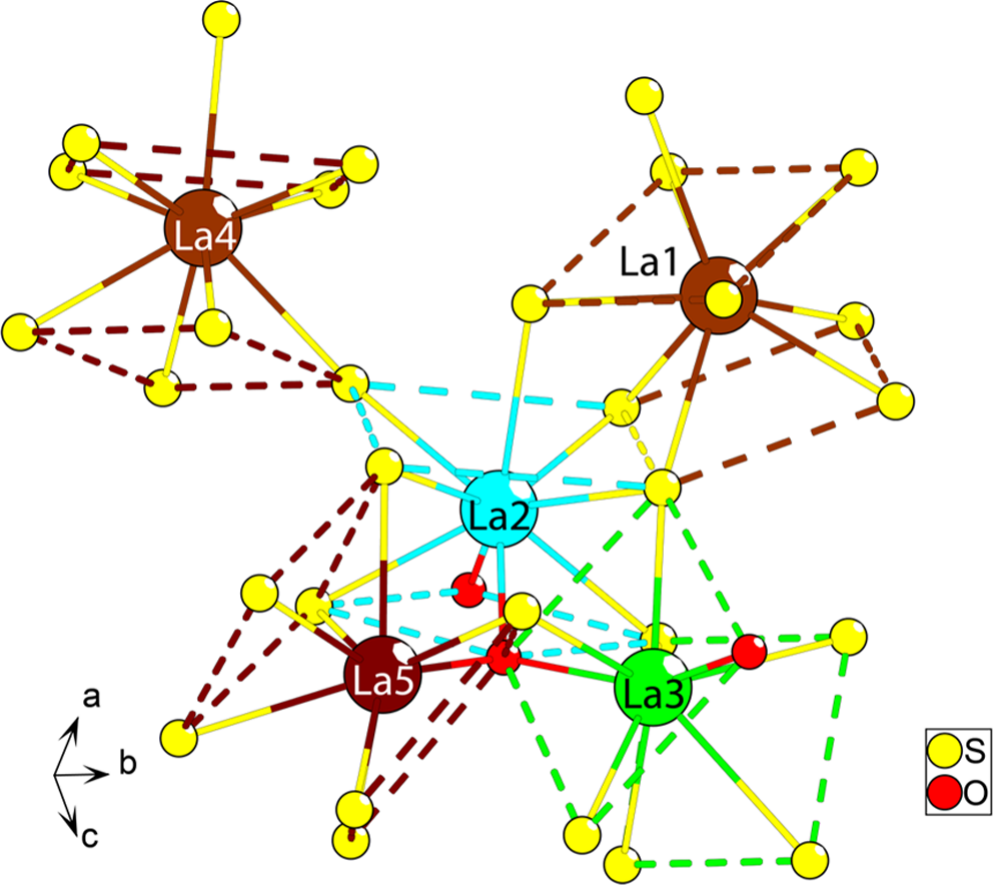
Five unique lanthanum coordinations of the LTCSO crystal structure. The sulfur and oxygen are yellow and red, respectively. The dashed bonds are to clarify the orientation of the square antiprismatic coordination (the cap is not represented like this) of each lanthanum color-coded for their respective lanthanum. The yellow dashed bond is shared between the two such coordinations. The disordered sulfide occupation is omitted for clarity.

The lanthanum positions exhibit several different coordinations within the LTCSO structure, including both homoleptic and heteroleptic arrangements (Figure 3). Every lanthanum position exhibits a capped square antiprismatic coordination. Two of the lanthanum positions are homoleptic (Figure 3, La1 and La4), while the remaining three are heteroleptic, each with distinct coordination. Two positions exhibit a 2 + 7 heteroleptic arrangement (Figure 3, La2 and La3), where the oxide substitutions for La2 and La3 are arranged in a *trans*-configuration on the opposite and same faces as the cap coordinations, respectively. The final position exhibits a 1 + 8 configuration (Figure 3, La5), where the substituted position is on the same face as the cap coordination.

**Figure 4:**
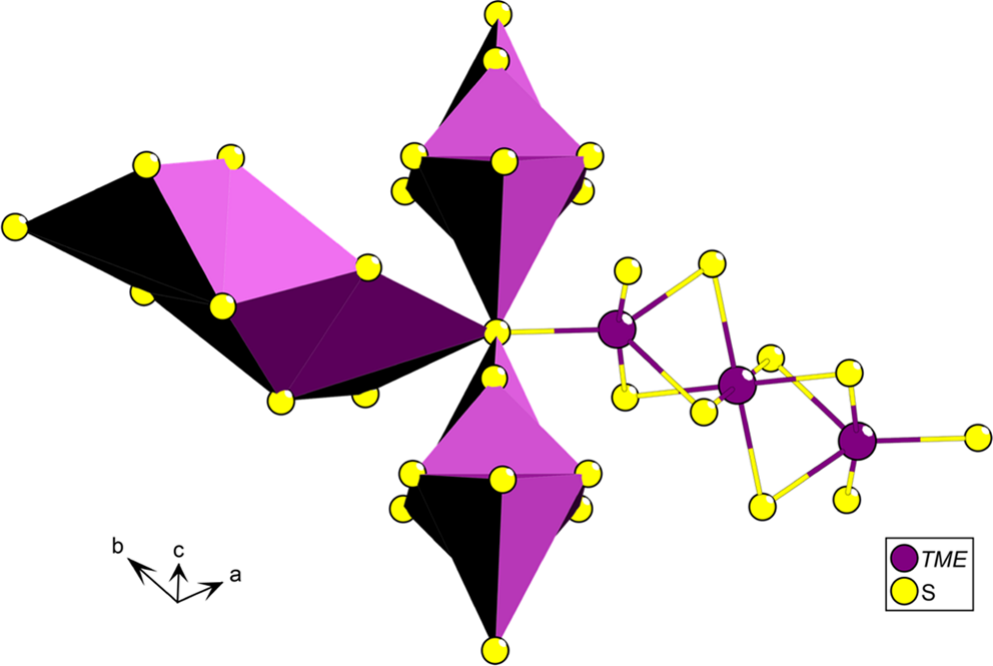
Structure and interconnection of the *TME* triplets in the LTCSO structure.

The *TME* triplets consist of two different coordinations: The central *TME* position is octahedral, while the terminal positions are trigonal bipyramids (Figure 4). The face-sharing of the three polyhedra constitutes a triplet, and four triplets share a common, inversion symmetric, sulfide coordination. This arrangement occurs at each triplet terminus, where the terminal *TME* positions are arranged into a planar arrangement, forming a slightly distorted square arrangement around the sulfide position. The shared sulfide positions arrange the triplets into 1D chains along the *b*-axis. By the same sulfide position, these chains are connected along the *a*-axis as well, although with a step between each chain, forming a 2D planar structure with stair-like steps. It may be noted that the trigonal bipyramidal coordinations are slightly distorted such that the *TME* position is slightly shifted toward the adjacent inversion symmetric sulfide position. This distortion is slightly more pronounced in the Cr analogue; the Fe analogue is closer to a regular structure, but the distortion is still present.

Closely situated, tetrahedrally coordinated copper positions are observed as half occupied, which is interpreted as structural disorder. The occupancy is split between two adjacent positions related by a mirror symmetry with a disordered sulfide position in between. The disordered sulfide position has no distinct point maxima observable in the LCCSO SC-XRD data. The density is distributed around an elongated tube, roughly corresponding to an equidistant arrangement from one of the two potential copper positions.

Whether there is long-range order to this arrangement cannot be deduced from the data at hand, it is, however, most certain that the two positions cannot be simultaneously occupied. From a basic consideration of interatomic distances, the sulfide disorder is simply a consequence of the Cu ion repulsing the sulfide ion away from the mirror plane position centered between the Cu positions. Removing the split occupancy by refining the data under the lower symmetry space groups was attempted, but this still resulted in a 0.5 occupancy; a single full site occupancy significantly worsened the refinement.

### Powder X-ray Diffraction (PXRD)

**Figure 5:**
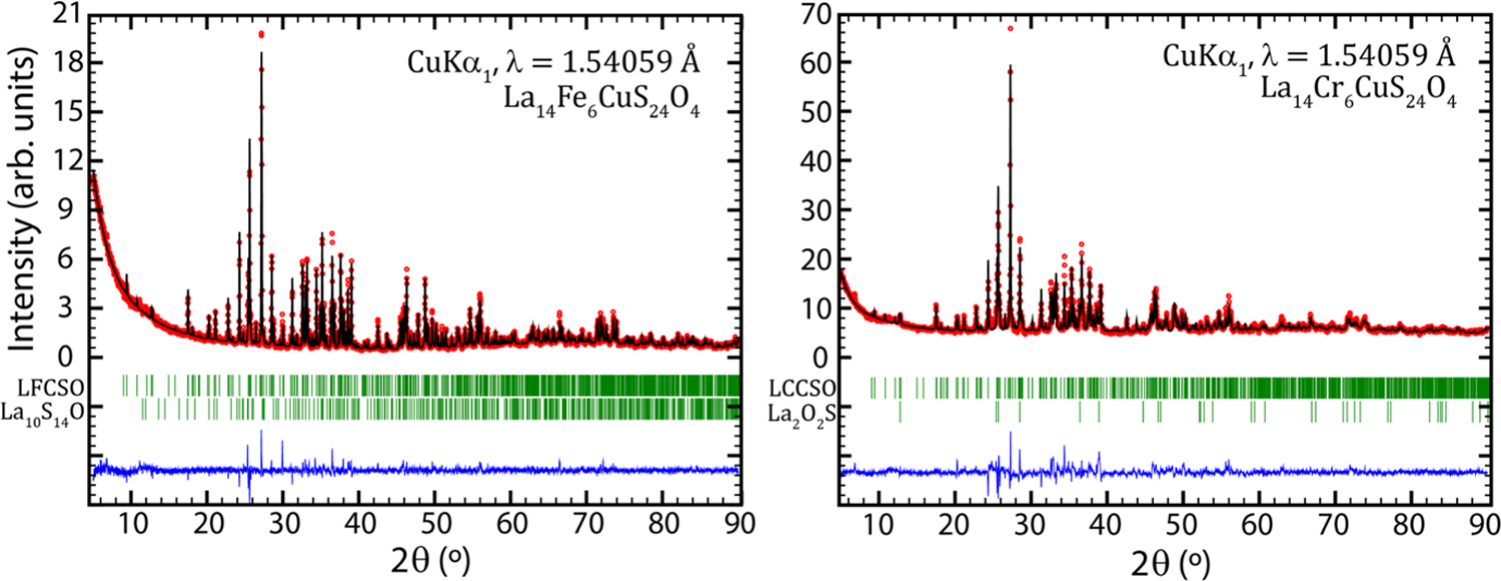
Rietveld refinements of LFCSO and LCCSO.

To improve the accuracy of the lattice parameter determination, PXRD was utilized. Refining the lattice parameters from a pure sample, shown in Figure 5, the obtained lattice parameters for LCCSO and LFCSO are *a* = 15.3853(5) Å, *b* = 13.9729(5) Å, *c* = 10.5074(4) Å, and β = 116.227(3)° and *a* = 15.4303(2) Å, *b* = 14.0033(2) Å, *c* = 10.4909(2) Å, and β = 116.261(2)°, respectively. The fitting largely indicates that the structure is correct, and the compounds are predominantly a single phase, although there are secondary phases, such as La_2_O_2_S,^11^ and traces of La_10_S_14.5_O_0.5_^12^ in LCCSO. In LFCSO, there is also some La_10_S_14.5_O_0.5_ present, as is nearly always the case in lanthanum oxysulfide synthesis, as well as at least one unidentified phase, and likely some presence of the decomposition product.

Notably, the Cr analogue is significantly less crystalline than Fe; there are multiple peaks in the LCCSO PXRD which should be distinct, as seen in the LFCSO equivalent, which considerably overlap with each other.

### Scanning Electron Microscopy (SEM) and Energy-Dispersive X-ray Spectroscopy (EDX) Analysis

**Figure 6:**
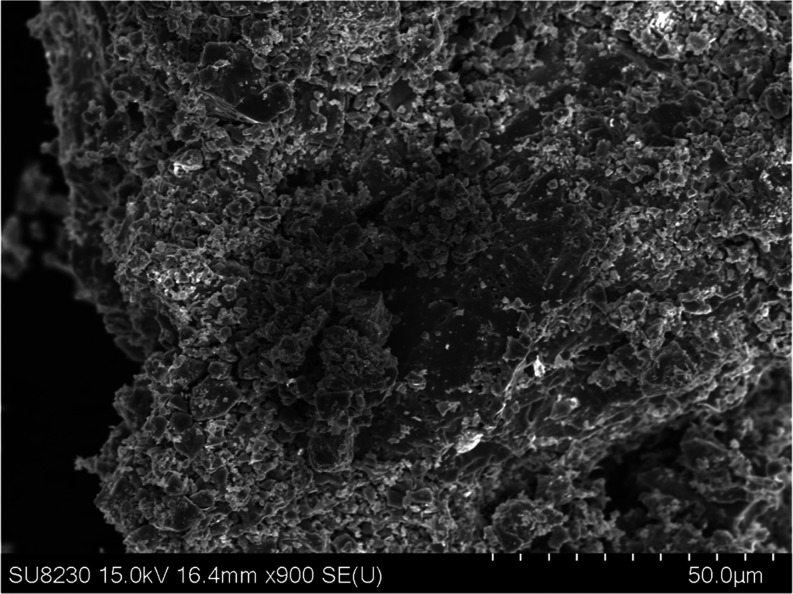
Small and loosely connected crystallites of LCCSO. The dark spot in the center of the image changed color after the center of the area was exposed to the electron beam.

Viewed in SEM, the crystallites of LCCSO are notably very small and coarsely interconnected. It is difficult to make out any distinctive habitus for the compound (Figure 6). A notable aspect is that the compound appears to be sensitive to the electron beam, with regions of the compound exposed to the beam darkening in the images. This darkening effect occurs in a rather broad area, even if the electron beam is only targeted at a single point in the center, which could be attributed to charging-up effects caused by poor conductivity of the sample, but this does not match with the measured electric resistivity of LCCSO. Alternatively, the compound could be unstable against the electron beam, but the large area affected with only a single central position being exposed to the beam would be unusual.

The composition of LCCSO, as determined by EDX analysis, is given in Table 2.

**Table 2:** Composition of LCCSO, as Determined by EDX Analysis

element	La	Cr	Cu	S
SC-XRD	14	6	1	24
composition	14.0(7)	5.9(5)	1.6(5)	24.1(8)

The determined composition shows good agreement with the nominal stoichiometry of the target phase. All elements, except Cu, have the target composition within the range of error. Cu, being the least prevalent element, has the greatest relative error, and the EDX measurement cannot determine whether the Cu positions are half or fully occupied.

During the analysis, a single crystallite of a secondary phase was observed, namely, La_2_O_2_S, which was also observed in the PXRD analysis. No other phases were observed.

**Figure 7:**
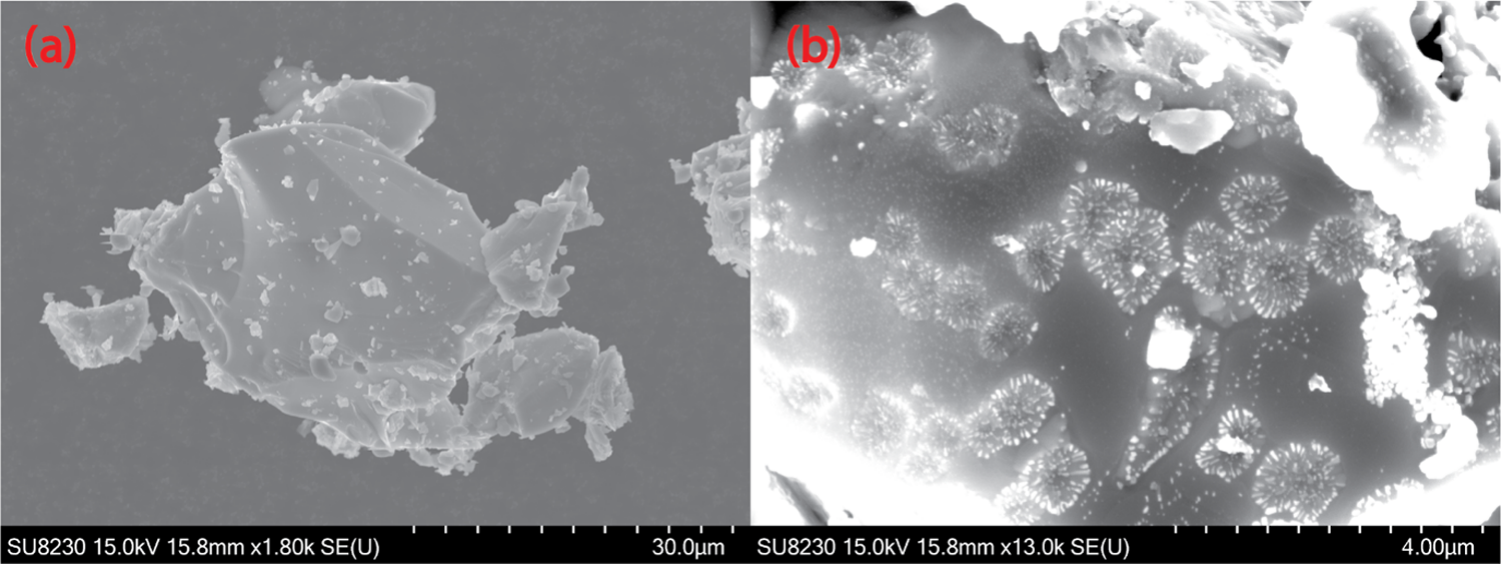
(a) SEM image of an LFCSO crystal. (b) SEM image of the distinct decomposition patterns that occurred with certain crystals of LFCSO upon exposure to the electron beam.

Compared with LCCSO, the crystals of LFCSO are considerably larger, with fewer of the small fragments so prevalent in the LCCSO image (Figure 7a). There is also a notable lack of habitus, with the crystals assuming a wide range of structures. It was noted that the LFCSO was unstable under the electron beam, resulting in decomposition. Some crystals exhibited distinct decomposition behavior, illustrated in Figure 7b. Small distortions on the surface, in roughly circular dispersion around select points. Notably, the center of these decomposition patterns does not correspond to a point where the electron beam was particularly focused, but rather appears to be randomly distributed around the crystal hit by the beam.

The composition of LFCSO, as determined by EDX, is given in Table 3:

**Table 3:** Composition of LFCSO, as Determined by EDX Analysis

element	La	Fe	Cu	S
SC-XRD	14	6	1	24
composition	14.0(3)	6.5(4)	1.2(2)	22.7(5)

All elements except sulfur show decent agreement with the expected stoichiometry. The sulfur composition is slightly lower, but as it is the lightest element, the expected error is also the greatest.

### Magnetism

**Figure 8:**
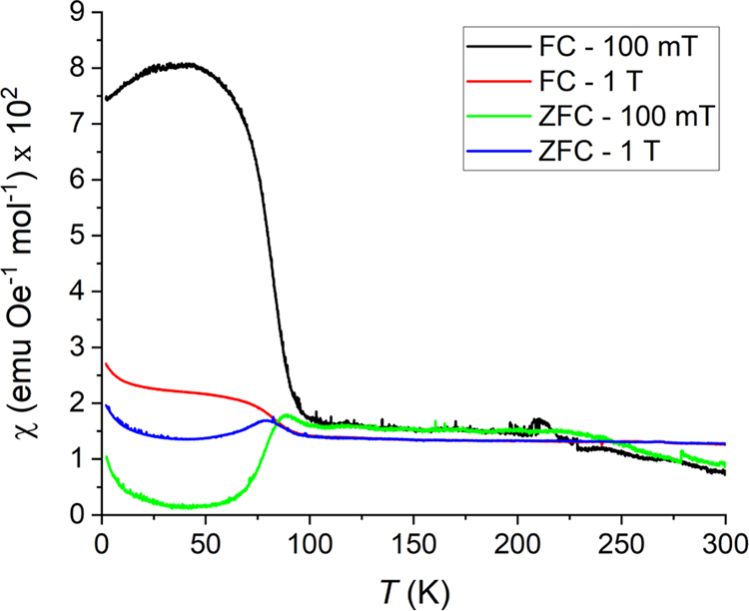
Direct current (DC) magnetic susceptibility of LCCSO across the 2–300 K temperature range with applied fields of 100 mT and 1 T.

LCCSO does not appear to exhibit Curie paramagnetism in the 2–300 K range (Figure 8). Starting at about 100 K, there is a change in the magnetic signal. Above this spin-freezing temperature, field-cooled (FC) and zero-field-cooled (ZFC) overlap, but below, they exhibit substantial differences. ZFC data show what could appear as a comparatively broad AFM spin-freezing region after an initial increase in susceptibility, while the FC curves show what appears to be an FM transition. Similar behavior in a static magnetic field is observed in, for instance, certain perovskites such as SrRuO_3_^13^ and La_0.5_Sr_0.5_CoO_3_.^14^ The formation of magnetic domains could be a feature of either a ferro- or a ferrimagnetic material at low fields, but it could potentially also be observed for a spin-glass, a mictomagnet, or similar.

The susceptibility at higher temperatures is nearly temperature-independent, appearing linear. The origin of this behavior is unknown; it resembles what one could expect from a metallic phase but could potentially originate from impurities.

**Figure 9:**
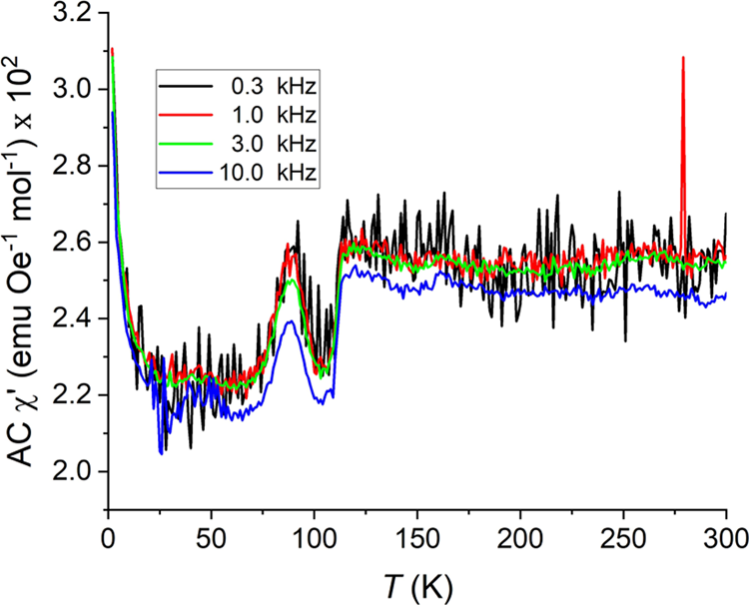
Real component of the AC susceptibility of LCCSO in the temperature range of 2–300 K.

The real component alternating current (AC) susceptibility data exhibit two features of note (Figure 9). The first is a discontinuous shift in the susceptibility at 114 K. The nature of this transition is unknown, but physical interpretation of AC susceptibility in general would suggest an electron redistribution at this temperature.

The second feature occurs at the same temperature as the broad transition in the FC DC measurements; the AC susceptibility exhibits a broad peak. The temperature of the broad AC peak appears to be nearly independent of the AC frequency. This would imply that the turning of the magnetic domains in LCCSO involves a large activation energy, approaching a full magnetic ordering. The 100 mT ZFC and 1 T FC curves exhibit a second anomaly at around 30 K, where the susceptibility increases; this is assumed to originate from paramagnetic secondary phases.

The magnitude of the imaginary component of the AC susceptibility is about 5% of the real component, which suggests a comparatively small domain size, in diametric contrast to the large domain size, which would be associated with the invariance of the AC susceptibility to the magnetic field frequency.

Magnetization measurements of the low-temperature state of LCCSO show that the total spin, causing the FC curve to appear very different from the ZFC data in Figure 8, is very low, about 0.2 μ_B_ mol^–1^ with an applied field of 5 T. It may be assumed that the low-temperature magnetic state does not correspond to a classical ground state.

**Figure 10:**
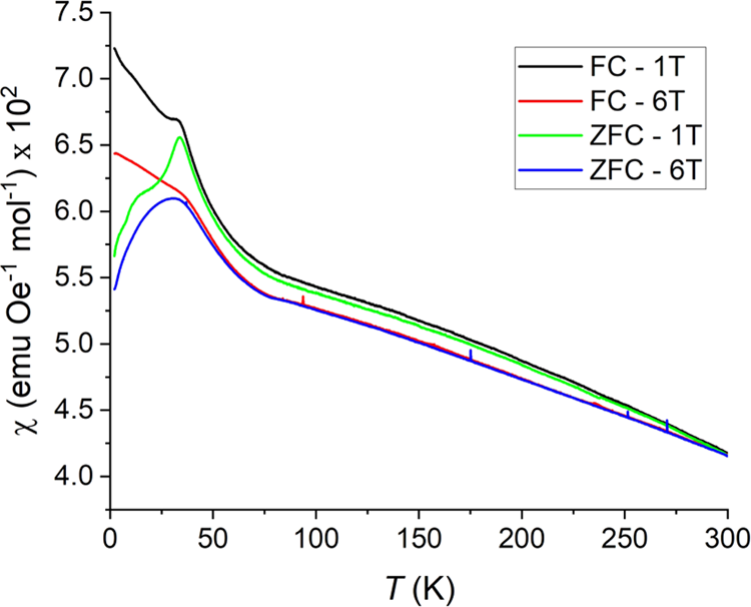
FC and ZFC DC susceptibilities of LFCSO at 1 and 6 T.

LFCSO, on the other hand, exhibits markedly different behavior (Figure 10). While it exhibits a similar ZFC transition, the susceptibility behavior is similar to an AFM ordering below this temperature, rather than the susceptibility leveling off like LCCSO. Additionally, there is a second transition around 20 K where the similar behavior to LCCSO transitions to exhibiting regular AFM behavior. This last feature is smeared away in stronger applied fields, resulting in one broad transition instead.

At higher fields, and above about 80 K, LFCSO exhibits what appears to be closer to a paramagnetic state. It is distinctly not, however, as the second derivative of the susceptibility with temperature is negative throughout the full range. Same as with LCCSO, the origin of this behavior is unknown. It could be due to impurities in the sample, but as both compounds exhibit something similar, there is also a case to make the contribution is intrinsic.

The most significant departure from the characteristics of LCCSO is the lack of a highly magnetized state below the magnetic domain formation temperature in the FC measurements. The magnetic state is clearly changed from the ZFC measurement, but qualitatively, the measurement is more similar to the 100 mT ZFC measurement of LCCSO than the FC equivalent. Rather than a susceptibility indicative of a ferro- or ferrimagnetic state, the FC measurements are more indicative of a spin-glass or possibly a semi-spin-glass. It is also possible that the FC behavior corresponds to a broader ferrimagnetic transition that does not complete within the measured temperature range.

Magnetization measurements revealed some degree of ferromagnetic impurities in both compounds; however, these were comparatively minor. Assuming elemental iron as the source of the ferromagnetic signal in both the Cr and Fe samples, there is 0.03 wt % or less of this impurity in either sample. Furthermore, as the qualitative behavior of the compounds’ DC susceptibility remains consistent with different applied fields and the two compounds exhibit similar behavior despite different magnetic ions, it is highly plausible that the observed properties are intrinsic to the compounds themselves.

### Heat Capacity

**Figure 11:**
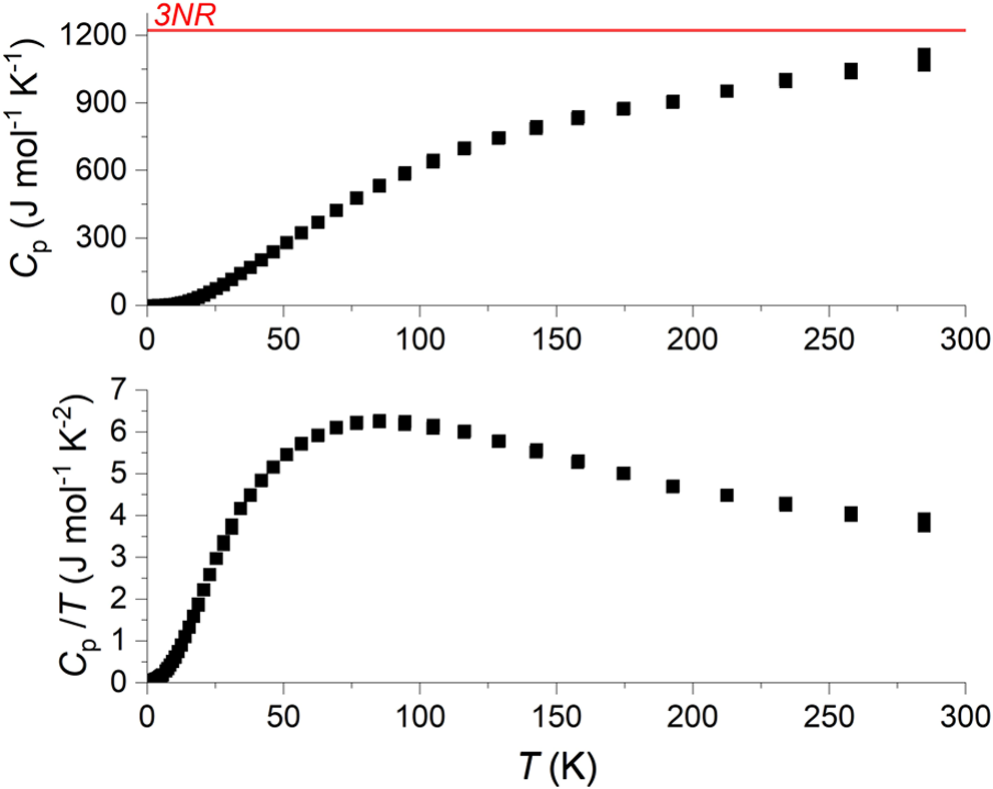
Heat capacity measurement of the LCCSO.

The heat capacity of LCCSO, as well as the heat capacity divided by the temperature, is shown in Figure 11. First, there is no indication of the compound undergoing any distinct transition across the measured temperature range. Neither the sharp transition in the AC susceptibility nor the onset of the high-magnetization state appear to be associated with any obvious change in entropy. As such, we may rule out the option of LCCSO being a ferrimagnet, or other ordered magnetic phase.

### Electrical Properties

**Figure 12:**
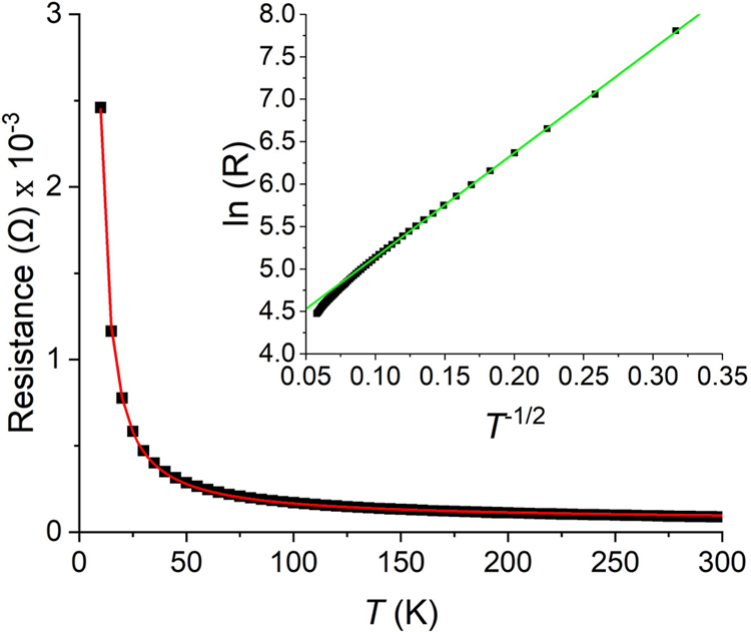
Electrical resistance of LCCSO, measured by a two-point contact approach. The black points are the measured data points, and the red line is the fitting of the Mott variable-range hopping model. The green line is a linear fit to the natural logarithm of the resistivity against the inverse square root of the temperature.

The electrical resistance of LCCSO across the 10–300 K range (Figure 12) showed that the compound behaved like an insulator across the full temperature range. No distinct change in the properties occurred around the temperatures that showed clear transitions in the magnetic measurements. As such, we may establish that the temperature-independent behavior of the LCCSO susceptibility was not due to the compound assuming a metallic state, and the sharp transition in the AC measurement does not correspond with a charge ordering mechanism. This leaves the nature of these magnetic features unknown, as far as the authors are aware.

The resistivity appears to be controlled by a variable-range hopping mechanism. Fitting the data to the Mott variable hopping equation across the full temperature range gives a dimensionality of approximately 1. This fit does exhibit systematic deviations in the high-temperature regime; from about 55 K and above, one obtains a better fit with a dimensionality of 3. This makes intuitive sense when considering the crystal structure, which exhibits several variations of 1D substructures. The higher dimensionality at higher temperatures simply corresponds to the electrons having sufficient energy to conduct along less favorable axes. For the full temperature range fitting, the parameters are *R*_0_ = 45(2) Ω, *T*_0_ = 171(9) K, and *d* = 1.05(3).

### Density Functional Theory (DFT)

Due to the unknown nature of the disorder in the structures, the DFT calculations are necessarily based on simplified and idealized structures. Whether there is a superstructure or whether the structural elements are fully disordered is unclear; thus, the results described here should be considered cursory.

**Figure 13:**
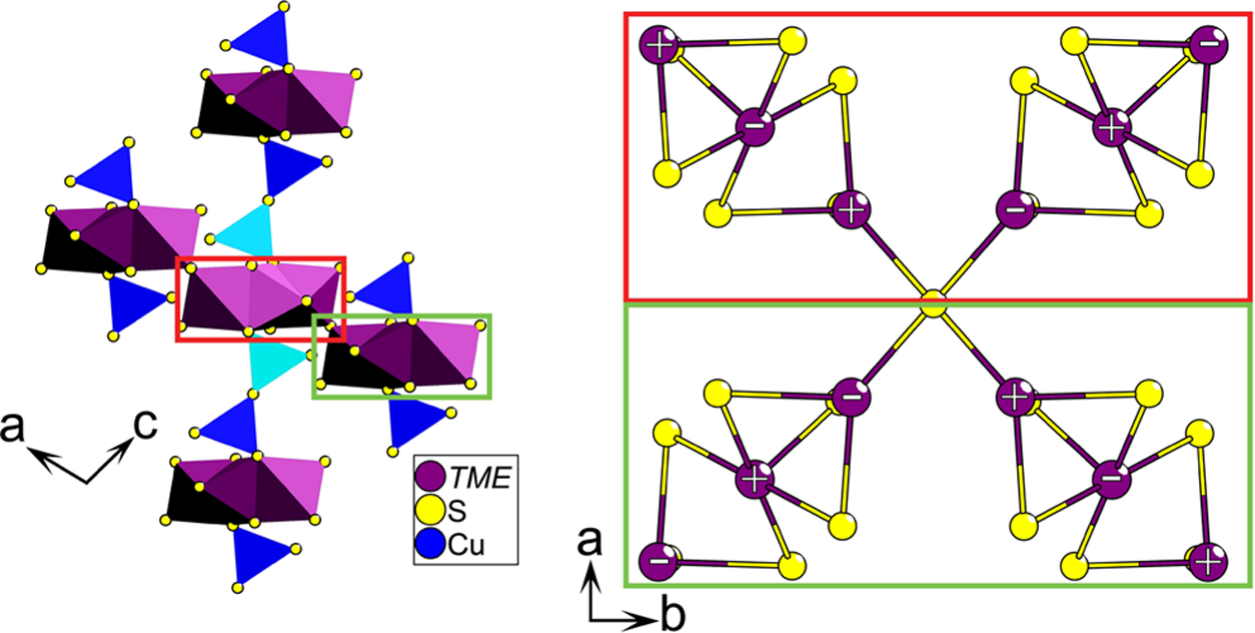
Most energetically favorable Cu ordering for the LTCSO structure along with the most energetically favorable magnetic configuration. The light blue tetrahedra indicate unoccupied copper sites. The colored boxes indicate corresponding parts of the crystal structure. The copper sites adjacent to a given TME chain, parallel with the *b*-axis, are either fully occupied or fully unoccupied. The plus and minus signs indicate the relative direction of spin.

The predicted ground magnetic structure of LTCSO, shown in Figure 13, was found to be the same for all configurations of the structure. Each trimer arrangement of the transition metals assumes a linear AFM arrangement, where the terminal positions assume parallel spin, and the central position is oppositely arranged. In effect, each trimer forms a small ferrimagnetic unit. These ferrimagnetic units are then arranged in an antiparallel configuration between the adjacent units along the *b*-axis. Across the vertices where four ferrimagnetic units intersect, the magnetic alignment is antiparallel with any closest two units and thus necessarily parallel with the oppositely situated unit (Figure 13). While the intertrimer interactions constitute the strongest couplings in the system, the intratrimer interactions are of comparable magnitude. As such, it is unlikely the system assumes a state where the trimers remain internally ordered, while aligning freely relative to the adjacent units. Adjacent trimers along the *c*-axis, i.e., adjacent trimers in different 2D layers, were predicted to preferentially align with their spins in parallel.

The lowest-energy configuration of ordered Cu-occupation that could be achieved for LTCSO within a 2 × 2 × 2 supercell arranged all Cu positions into a fully occupied ordering adjacent to every alternate step of the spin chain (Figure 13). Every successive step of the 2D plane thus alternates between full and zero occupancy of the adjacent Cu sites. The effective space group for this arrangement is *P*2_1_/*m*.

Nominally, to attain charge neutrality, the transition metal cations of the LTCSO structure must have a formal oxidation state averaging 2^1/6^. Alternatively, this may be considered as five divalent and one trivalent TME ion, or four divalent and two sites with a valence of 2^1/2^. For LTCSO, the DFT calculations establish the latter two descriptions to be the more appropriate by analysis of the magnetic moments associated with each position, as the oxidation states appear to be localized to the octahedral sites, albeit with varying degrees of specificity according to the copper occupation. Comparing the calculated magnetic moments for the divalent and trivalent species, Cr^2+^ and Cr^3+^ exhibit average magnetic moments of 3.53(±0.02) and 2.90(±0.01) μ_B_, respectively, showing clearly distinct magnetic states. The magnitude of the calculated values underestimates the expected moments, but this is typical of DFT. The difference between the calculated Fe^2+^ and Fe^3+^ magnetic moments is much smaller, 3.49(±0.02) and 3.79(±0.01) μ_B_, again significantly underestimating the theoretical values, but these values are typical of di- and trivalent iron coordinated with sulfur with *U*_eff_ = 3 eV, and the qualitative trend is correct.

For the regular sulfide arrangement of LCCSO, the trivalent Cr is the sole chromium position with a substantially lowered magnetic moment, as would be expected from Cr^3+^ relative to Cr^2+^. The other five chromium positions exhibit approximately identical magnetic moments, with the difference between them being about 2 orders of magnitude less than the Cr^2+^ – Cr^3+^ difference. The trivalent chromium sites are exclusively the octahedral sites; what octahedral sites are trivalent is controlled by the adjacent Cu occupancy and the local sulfide ordering. In the lowest-energy Cu configuration, with the regular sulfide arrangement, the rules are simple: A Cr position with an adjacent occupied Cu^+^ position will assume a divalent state, while a Cr position without an adjacent Cu^+^ occupation will assume a trivalent state. If the copper ordering does not exhibit sufficient octahedral sites without adjacent copper, the Cr sites with one adjacent Cu will assume a trivalent arrangement. If the copper ordering is such that all octahedral Cr sites have an identical environment, then the octahedral sites will assume a 2.5 valency. Note that among the tested calculation cells, the 2.5-valent state was the least favorable configuration. As such, one may interpret the results to suggest LCCSO is best described in terms of discrete oxidation states as La_14_Cr_5_^2+^Cr^3+^ CuS_24_O_4_.

In the case of regular LFCSO, the behavior was mostly identical: The most favorable configuration exhibits the same Cu arrangement and localized oxidation states. The principal difference is simply due to the trivalent Fe positions exhibiting an increased magnetic moment, in contrast to the Cr variation due to their electron configuration.

The irregular sulfide arrangement reduces the degeneracy of the system, in that the position of the Cu ion is shifted toward one of the two adjacent *TME* positions and away from the other, but the general magnetic configuration remains the same as the regular arrangement. The critical difference is that the distortions introduced by the irregular sulfide arrangement also affect the local oxidation states. Rather than the ordered sulfide arrangement of clearly defined rules for what octahedral site exhibits what Cr oxidation state, exceptions appear. Further, the states now converge to distinctly di- or trivalent, regardless of how the Cu occupancy is arranged, removing the partial states observed for the regular sulfide arrangement. A difference observed between the Cr and Fe analogues with respect to the sulfide arrangement is that the regular Cr analogue (a reminder, this is the structure with a fully ordered sulfur arrangement) is more favorable than the irregular, while conversely the irregular Fe analog is more favorable than the regular equivalent.

The disorder introduced into the arrangement of the different oxidation states from the irregular sulfides allows for an avenue by which the complete AFM arrangement may be broken and a sum magnetic moment may be achieved to explain the experimentally observed properties. To investigate this possibility, several structural and magnetic configurations of LCCSO and LFCSO with nonzero magnetic moments were tested, but these were found to be significantly less favorable than the zero-moment arrangements. Altering the + *U*_eff_ value for chromium did not allow for a ferrimagnetic state to be favorable compared with the full AFM. The most favorable ferrimagnetic states exhibited the same copper arrangements as the most favorable full AFM structure. This inability to achieve a stable ferrimagnetic state could, considering the experimentally observed AFM ordering of LFCSO at low fields, be considered an indication that the high-magnetization state is not the ground state of the LTCSO compounds but rather induced by the application of an external field.

The calculated band gap as well as the transition of LTCSO is found to be significantly dependent on the exact Cu lattice site occupancy. The sulfide disorder is less impactful for the LCCSO structure; while it does affect the magnitude of the band gap, the symmetry of the transition was not observed to be affected in the calculations, so long as the qualitative magnetic structure remains unchanged.

**Figure 14:**
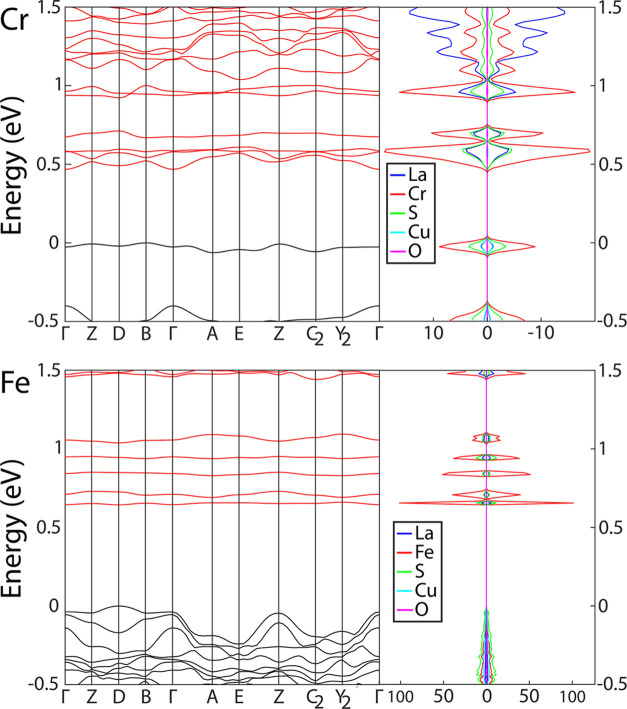
Band structure and DOS of regular LCCSO and LFCSO.

The band structure and DOS of both LCCSO and LFCSO (both regular) are listed in Figure 14. For the lowest-energy Cu configuration of regular LCCSO, the compound exhibits an indirect band gap with a width of 0.69 eV with distorted Cu-tetrahedra, or 0.47 eV with regular tetrahedra, with a *Z*-Γ transition. The valence band maximum (VBM) and the conduction band minimum (CBM) consist primarily of Cr-3d states, making LCCSO predominantly a Mott insulator. Specifically, the CBM consists of 3d states originating from the divalent chromium sites, both the trigonal bipyramidal and octahedral sites, while the VBM primarily consists of states originating from the trivalent, octahedrally coordinated chromium positions. Additionally, the VBM exhibits a smaller proportion of S-3p and Cu-3d states.

Regular LFCSO exhibits a different transition, with a direct band gap of 0.64 eV at the *D*-symmetry point. The VBM is primarily composed of S-2p states, with Cu-3d states as a secondary component. The CBM on the other hand is composed almost exclusively of Fe-3d states originating from trivalent, octahedral Fe, making LFCSO a charge-transfer insulator.

Again, it should be emphasized that whether this compound is intrinsically disordered is not known. The observable bulk properties of both compounds may well be from a range of disordered Cu- and S-arrangements. The different structures used in the calculation cells resulted in a wide range of options for properties such as the width of the band gap and the transition symmetry.

## Discussion

The differences in the magnetic properties between the two compounds may be explained in terms of the characteristics of the electron configurations: Fe^3+^ exhibits a d5 electron configuration, compared with the d3 configuration of Cr^3+^. The spherically symmetric d5 configuration exhibits less magnetocrystalline coupling than the asymmetric d3 equivalent and would thus be more susceptible to coercion by an external magnetic field. Conversely, the more strongly coupled d3 configuration would be more prone to assuming a single fixed magnetic configuration.

Considering the experimentally observed magnetic properties of both LCCSO and LFCSO, the correct categorization of the magnetic properties that they exhibit is somewhat complicated. While the magnetic behaviors of the two analogues are rather different, there are also similarities the two share.

The most coherent description of the properties of LTCSO would be as a freezing of spin domains, but ultimately, we can conclude that available data on the LTCSO phases are insufficient to make a definitive statement about the origin of the magnetic properties. Neutron data or Mössbauer spectroscopy to investigate the magnetic structure experimentally would be necessary and is a topic for further work with these compounds.

## Conclusions

Powder and single crystals of La_14_*TME*_6_CuS_24_O_4_ (*TME* = Cr, Fe) were obtained by mixing La, La_2_O_3_, *TME*, Cu, and S. The crystal structures were determined by SC-XRD and refined by PXRD. The compounds exhibit a novel structure described by the *C*2/*m* space group. The crystal structure exhibits transition element spin chains, resulting in the formation of spin domains in both analogues below temperatures of about 100 and 33.5 K for the Cr and Fe analogues, respectively. We lack the data to establish the nature of the magnetic properties with certainty, but they are believed to be nonclassical spin-domain compounds. The Cr analogue does not exhibit a first-order transition at any temperature, as indicated by heat capacity measurements. DFT calculations suggest that the ground-state magnetic structure of the compounds consists of ferrimagnetic triplets, interconnected in an AFM arrangement. Further, the calculation results suggest the formal oxidation states of the compounds may be described as La_14_*TME*_5_^2+^*TME*^3+^ CuS_24_O_4_ (*TME* = Cr, Fe).

## Experimental Section

### Sample Preparation

Prior to the completion of the syntheses, all handling of the compounds and their precursors took place within an argon-filled glovebox (H_2_O and O_2_ < 1 ppm). Mixtures, totaling about 0.5 g each, with nominal stoichiometric compositions La_14_*TME*_6_CuS_24_O_4_ (*TME* = Cr, Fe) were weighed out consisting of La filings (Thermo Scientific, 99.9%), La_2_O_3_ powder (Molycorp, 99.99%), Fe powder (Alfa Aesar, 99%), Cr powder (Sigma-Aldrich, 99.5%), Cu powder (>99%), and S chunks (>99%), which were crushed together and mixed in an agate pestle and mortar. An additional 10 w/% Cu was added to the Fe composition. The extra Cu is not assumed to be part of the stoichiometry, but to counter loss of Cu from the mixture during synthesis. Each homogeneous mixture was pressed into a 13 mm diameter pellet with 35 kN of force before breaking the resulting pellets into chunks fit in a corundum crucible. The filled crucible was inserted into a silica ampule, which was evacuated and sealed with an oxygen–hydrogen torch. The samples were heated in a muffle furnace. The LFCSO was initially heated with a 1 °C min^–1^ ramp to 400 °C, resting at this temperature for 5 h. Then, a second 5 °C min^–1^ ramp to 950 °C followed, resting again at this temperature for 48 h. LCCSO followed the same initial step, but the second rest temperature was 1000 °C and was left to sinter for 168 h with 2 intermediate regrinds. With each regrind, the furnace was turned off and left to cool at an ambient rate to room temperature. The cooled sample was returned to the glovebox, reground and repelletized before being sealed, and returned to the resting temperature with a heating ramp of 5 °C min^–1^.

A note on synthesis: the Fe analogue appears metastable at the synthesis temperature of 950 °C. At 900 °C, LFCSO was also confirmed to be metastable, but, at this temperature, the reaction time to obtain a decent product was sufficiently long that the decomposition product could form in considerable quantities. After the LFCSO forms, it starts to decompose by a mechanism suspected to be loss of a copper compound to sublimation, thus, the extra Cu in the synthesis composition. The decomposition product is an as yet unreported phase. The formation is faster than the decomposition; therefore, a relatively pure phase could still be obtained. Synthesis attempts below 900 °C failed to produce the target phase.

The single crystals used for the determination of the crystal structure were retrieved from the final product for the Fe analogue. The single crystal for the Cr analogue was retrieved from a synthesis with nominal composition La_14_Cr_4_Cu_2_S_23_O_4_, which was heated with a 5 °C min^–1^ ramp to 1000 °C, and left to rest for 24 h.

The products were both crystalline black materials, which appeared to be stable under ambient conditions. LCCSO was attempted to be sintered into a pellet, but the product pieces were notably fragile, highly porous, and challenging to handle, limiting the property investigations.

### X-ray Structure Determination

Single-crystal (SC-XRD) data were gathered using a BRUKER D8 Venture single-crystal diffractometer, utilizing a Mo Kα InCoatec microfocus X-ray source and a Photon 100 detector. Powder X-ray (PXRD) data were obtained from a BRUKER D8 Discover, using a Bragg–Brentano geometry, a Ge(111) Johanssen monochromator, Cu Kα_1_ X-rays, and a Lynxeye detector. The sample holder was a zero-background-oriented silicon plate covered in a minimal layer of silicon grease. The structure solution and refinement were successful by using the JANA2020 software.^15^

### Physical Property Measurement System (PPMS)

Magnetic and heat capacity measurements were carried out using a Quantum Design PPMS. Heat capacity measurements were performed between 2 and 300 K on a cold-pressed, polycrystalline sample, with the nonadiabatic thermal relaxation method. The sample was equilibrated for 5 min at each temperature before measuring. The sample coupling remained above 90% throughout the investigation. The DC magnetic measurements were carried out on a sample from a powdered pellet in a polypropylene sample holder. Field-cooled (FC) and zero-field-cooled (ZFC) measurements were carried out in the range 2–300 K, utilizing applied magnetic fields of 100 mT and 1 T. The AC susceptibility was measured using a 5 Oe AC field, with frequencies ranging from 300 Hz to 10 kHz.

Electrical resistivity measurements for LCCSO utilized the PPMS to control the temperature, but the resistance across the sample was measured using a MASTECH MAS830L multimeter with a two-contact approach between 10 and 300 K. The contacts were connected to the sintered pellet sample by silver-paint. The measurements were carried out during a constant ramping of the temperature of 5 K min^–1^, both up and down, with the average values between the two sweeps being used.

### Scanning Electron Microscopy (SEM) and Energy-Dispersive X-ray (EDX) Analysis

SEM imaging and EDX analysis were carried out with a Hitachi SU8230 field emission scanning electron microscope with an XFlash 6|10 EDX detector. The acceleration voltage was set to 15 keV for both imaging and elemental analysis. The EDX composition determination was based on elemental analysis of 38 and 22 different crystallites for LCCSO and LFCSO, respectively. The resulting compositions were averaged and normalized according to the stoichiometric lanthanum content. The oxygen content was not determined due to the accuracy of the EDX determination for the lighter elements being too low.

### Density Functional Theory (DFT)

DFT was utilized to investigate the compound’s electronic properties and magnetic arrangements. Further, as the experimentally determined crystal structures exhibited slight disorder, the calculations sought to determine whether there are local atomic arrangements with relatively low energy that were not discernible in SC-XRD.

The calculations were carried out with the Vienna Ab initio Simulation Package (VASP),^16,17^ employing the generalized gradient approximation (GGA) Perdew–Burke–Ernzerhof (PBE)^18^ functional for the exchange-correlation energy. The calculations use projected augmented-wave (PAW) pseudopotentials,^19^ with a plane-wave energy cutoff of 500 eV. The self-consistent-field energy and ionic relaxation convergence criteria were set to 10^–6^ eV, and the forces on all atoms were less than 0.01 eV Å^–1^, respectively. The Hubbards + *U* approach, under the rotationally invariant Dudarev approach,^20^ was utilized to account for the strong d-orbital correlations, with the values set to +2 and +3 eV for the Cr- and Fe-3d orbitals, respectively. The values used were selected by referencing previous work from the literature.^21−23^ Five symmetrically distinct ordered arrangements of Cu occupancies that could be modeled with a 2 × 2 × 2 supercell were modeled out, and the reduced unit cells of these five structures were determined. These five reduced cells (or their supercells, if necessary, to represent the magnetic structure) were used as the calculation cells to determine the most favorable configuration, which was subsequently utilized for DOS and band-structure calculations. Two different variations of this structure were used to distinguish between sulfide arrangements. One arrangement places Cu positions in a regular tetrahedral coordination, while the other causes a distorted arrangement, which will henceforth be referred to as “regular” and “distorted” sulfide structures, respectively.

The sampling of the Brillouin zone during the structural relaxation utilized a Γ-centered grid, with sampling grids (KPT grids) assigned for each reduced cell according to a density of 30 KPT Å^–1^. The final calculation cell uses a sampling grid of 3 × 2 × 2. The DOS calculations utilize a 9 × 6 × 6 grid. Integration over the Brillouin zone for all calculations of LTCSO (except the band structure) was carried out using the tetrahedron method with Blöchl corrections and a smearing width of 0.02 eV. The band structure calculations, as well as the LFCSO ionic relaxation with disordered sulfide structure, utilized Gaussian smearing with the same width. Both the lattice parameters and ionic positions were allowed to relax. The symmetry paths for the band structure calculations were determined with the assistance of the SeeK-path tool.^24,25^

## Supporting Information Available

Single-crystal structure refinements (LCCSO.cif) (CIF)
Single-crystal structure refinements (LFCSO.cif) (CIF)

## Supplementary Material

ao4c00564_si_001.cif

ao4c00564_si_002.cif
